# Association between telomere length and erectile dysfunction: a cross-sectional study

**DOI:** 10.3389/fendo.2024.1391013

**Published:** 2024-07-11

**Authors:** Xiaobao Chen, Binhong Liu, Junkai Zhou, Junwei Lin, Wei Jiang, Ruoyun Xie

**Affiliations:** Department of Urology, Fujian Medical University Union Hospital, Fuzhou, China

**Keywords:** telomere length, erectile dysfunction, national health and nutrition examination survey, cross-sectional study, sexual dysfunction

## Abstract

**Background:**

Leukocyte telomere length (LTL) serves as a significant biomarker of aging. Erectile dysfunction (ED) is a commonly observed condition among middle-aged and older men. The objective of this study is to explore the potential association between LTL and ED.

**Methods:**

We utilized data from the National Health and Nutrition Examination Survey (NHANES) to examine the association between LTL and ED. Weighted multivariate regression analyses were performed as the primary statistical method. Subgroup analyses were conducted to investigate specific population subsets, and restricted cubic spline (RCS) analyses were employed to assess the non-linear relationship between LTL and ED.

**Results:**

The results of weighted multivariate regression analyses revealed a negative correlation between LTL and the risk of ED. Individuals with ED exhibited shorter LTL compared to those without ED. For each unit increase in LTL, there was a 54% reduction in the risk of ED (odds ratios[OR] 0.46, 95% confidence intervals[CI] 0.25-0.85). When LTL was considered as a categorical variable, the group with the longest LTL (Q5) had a 44% lower risk of ED compared to the group with the shortest LTL(Q1) (OR 0.56, 95% CI 0.39-0.81). A non-linear relationship was observed between TL and ED. Various sensitivity analyses were conducted to validate the stability of the results, and consistent findings were obtained.

**Conclusion:**

The negative association between leukocyte LTL and ED suggests that delaying the shortening of LTL may decrease the risk of ED.

## Introduction

Erectile dysfunction is a frequently observed disorder characterized by the persistent inability to achieve or maintain a satisfactory penile erection necessary for a fulfilling sexual experience (1). It primarily affects males over the age of 40 (2), data from the Massachusetts Male Aging Study revealed that degrees of ED, from mild to severe, affected 52% of males aged between 40 and 70 (3). The etiology of ED is multifaceted, involving both psychogenic and organic factors (4). ED has a strong correlation with a range of risk factors and comorbidities, including but not limited to obesity, lack of physical activity, tobacco use, alcohol addiction, diabetes, hypertension, dyslipidemia, and hypogonadism. Additionally, it is worth noting that ED can serve as a prognostic marker for cardiovascular disorders such as CVD, coronary heart disease, and stroke (5, 6). Identifying modifiable risk factors that contribute to this condition is pivotal, considering the substantial influence of ED on the health-related quality of life among aging men. This identification will facilitate the development of effective strategies for prevention and management. A comprehensive understanding of the intricate connections between ED and its associated risk factors is essential for mitigating the consequences of this condition on affected individuals and society at large.

Telomeres, which are evolutionarily conserved nucleoprotein structures found at the ends of chromosomes, play a crucial role as aging markers by regulating cellular senescence (7). As cells divide, the protective telomeric repeats undergo a natural shortening process. When telomeres shorten beyond a crucial length, cells halt their division and either enter a state of senescence or experience programmed cell death. Factors (8–12) such as chronic inflammation, oxidative stress, unhealthy lifestyles, and nutrition can accelerate this shortening, leading to adverse health conditions (13–16) such as infection, cardiovascular disease, cancer, mental illness, and other age-related disorders. Hence, comprehending the primary factors attributing to telomere diminution is imperative in unraveling the underlying pathophysiological mechanisms accountable for these enduring ailments.

Thus far, there exists a scarcity of research investigating the potential link between LTL and ED. To expand our comprehension of the influence of LTL on ED and offer valuable perspectives for its prevention, this cross-sectional investigation endeavors to explore the correlation between LTL and ED in a cohort of American adults. The data utilized for this study is extracted from the NHANES.

## Materials and methods

### Study population

This research utilized data from the NHANES conducted between 2001 and 2002. The study’s participants were individuals who possessed complete data on ED and LTL. Trained examiners carried out comprehensive family interviews to gather pertinent information, including demographic details, educational background, and personal medical history. Participants with incomplete demographic information, clinical outcomes, or laboratory data were not included in the study, resulting in a final sample size of 1,694 individuals aged 20 years and above. The study obtained ethical approval and acquired consent from the Ethics Review Committee of the National Health Statistics Center, which was communicated to all participants. Regarding data collection and definition, thorough procedures were implemented.

### Assessment of ED and LTL

Participant interviews were conducted in designated chambers at the Mobile Examination Center (MEC) during the NHANES survey to ensure privacy. To assess ED, an audio computer-assisted self-interview (ACASI) method was utilized. The questionnaire included a single adapted question from the Massachusetts Male Aging Study (17). Participants were requested to describe their ability to achieve and maintain a satisfactory erection for sexual intercourse. Response options included “always or almost always able”, “usually able”, “sometimes able”, and “never able”. For this analysis, individuals reporting “sometimes able” or “never able” to sustain an erection were classified as having ED. Conversely, respondents indicating they were “always or almost always able” or “usually able” were categorized as not having ED. Additionally, a sensitivity analysis was conducted using a more stringent criterion, defining ED (18) as individuals who reported being “never able” to maintain an erection. These categorizations allowed for the examination of varying levels of ED within the study cohort.

In our study, LTL was measured using the method described by Cawthon and Needham (19, 20). Peripheral blood was extracted and stored at -80°C with a concentration exceeding 100 ng/ml using phenol chloroform. Subsequently, quantitative real-time polymerase chain reaction (qRT-PCR) technology was employed. The T/S ratio (Ct (telomere assay)/Ct (single copy gene assay)), where Ct represents the number of cycles required to reach the threshold fluorescence level during qRT-PCR, was used to evaluate the relative LTL. The accuracy and reliability of TL data were ensured through a quality control review conducted by the Centers for Disease Control before linking it to the NHANES data files. The LTL in this study was defined as telomere length/standard reference DNA(T/S ratio). For further information on LTL, see the NHANES 2001-2002 data at https://wwwn.cdc.gov/Nchs/Nhanes/2001-2002/TELO_B.htm.

### Additional covariates of interest

To collect data on various factors, including age, marital status, race, Body Mass Index (BMI), education level, family income, physical activity, smoking status, alcohol consumption, and comorbid illnesses, standardized questionnaires were administered. Body composition was assessed using BMI, which is calculated by dividing weight by height squared (kg/m²). Race was categorized as non-Hispanic white, non-Hispanic black, Mexican American, or other. Education levels were classified as below high school or high school and above. Family income-to-poverty ratio was divided into three categories: =3.5, representing low, middle, and high income statuses, respectively. Smoking habits were classified as current, past, or never. Presence of pre-existing or co-existing conditions such as diabetes mellitus (DM), cardiovascular disease (CVD), anemia, chronic kidney disease (CKD), and hypertension was determined based on self-reported questionnaires. The Systemic Immune-Inflammatory Index (SII) is an index calculated using the counts of neutrophils, platelets, and lymphocytes in the peripheral blood. It is calculated using the following formula: SII = (platelet count × neutrophil count)/lymphocyte count.

### Statistical analysis

The NHANES analytical guidelines were followed for data processing. To account for NHANES’ complex sampling design, all analyses incorporated appropriate sample weights and strata. The “Survey” package in R was used to conduct weighted analyses. Weighted means ± standard error were calculated for continuous variables, and evaluated using either Student’s t-test or one-way ANOVA. Categorical data were presented as weighted percentages (standard error), and intergroup comparisons were made using the Chi-square test. A multivariate logistic regression model was performed to evaluate the independent association between LTL and ED, using weighted survey procedures. Non-linear relationships between LTL and ED were examined using adjusted RCS. Three models were utilized in our study: model 1 adjusted for age, race, marital status, FIR, BMI, and education level; model 2 built upon model 1 by adding adjustments for alcohol intake, smoking status, moderate activity, and vigorous activity; model 3 extended model 2 by including adjustments for anemia, hypertension, DM, CVD, CKD, CRP, AST, SII, and albumin. The results were reported as adjusted OR with their corresponding 95% CI. In our investigation, missing data was less than 10%, therefore no imputation technique was applied. The presence of multicollinearity was assessed using the variance inflation factor (VIF) method, with a VIF value of 5 or higher indicating multicollinearity. To ensure the robustness of our research outcomes, several sensitivity tests were conducted. Firstly, LTL was categorized into five equal groups as categorical variables, with the lowest quartile (Q1) serving as the reference category. This categorization allowed us to evaluate any discernible patterns in the relationship. Secondly, a subgroup analysis was performed by stratifying the data based on various factors such as age, race, marital status, education level, alcohol intake, smoking status, DM, CVD, CKD, and hypertension. An interaction test was employed to evaluate the heterogeneity of associations across different subgroups. Thirdly, a more stringent criterion for defining ED was applied in this study. Only participants who reported being “never able to get and maintain an erection sufficient for satisfactory intercourse” were classified as having ED.

The statistical analyses were conducted using R version 4.2.0. and Free Statistics software versions 1.7. A significance level of P < 0.05 (two-sided) was employed for all tests to determine statistical significance.

## Results

### Participant characteristics

**Figure 1:**
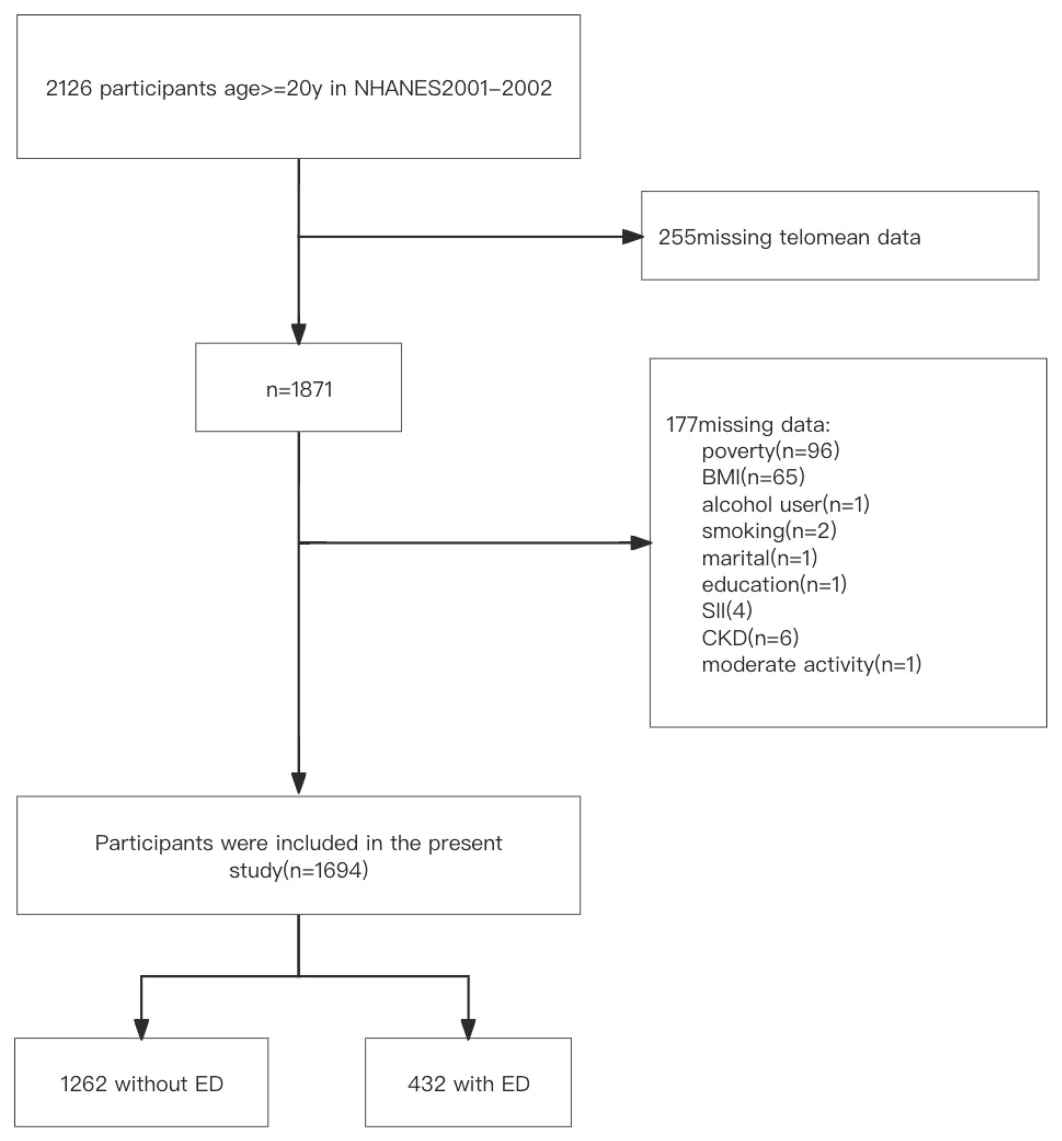
Flow diagram of the screening and enrollment of study participants.

Our investigation consisted of a total of 1,694 participants ranging in age from 20 to 85 years after excluding those with incomplete data. Of these individuals, 432 (25.5%) were identified as having ED, while 1,262 (74.5%) did not exhibit signs of ED (**Figure 1**). **Table 1** presents the baseline characteristics of the participants, categorized by their ED status. When comparing the non-ED group to the ED group, it was observed that the latter had a shorter LTL. Moreover, the ED group consisted of older individuals with lower levels of education and income. They also had a higher BMI, higher rates of smoking, engaged in less vigorous physical activity, and had a higher prevalence of hypertension, CVD, DM, CKD, and anemia. Additionally, participants in the ED group had lower levels of albumin and higher levels of CRP and SII.

**Table 1:** Baseline characteristics of participants, weighted.

Variable	Total	No ED	ED	P-value
Telomean	1.07 ± 0.02	1.09 ± 0.02	0.97 ± 0.02	< 0.0001
Age(years)	44.69 ± 0.58	41.49 ± 0.52	59.75 ± 0.80	< 0.0001
Age(years)				< 0.0001
<40	39.52(0.02)	45.64(2.05)	10.68(2.05)	
>=40	60.48(0.04)	54.36(2.05)	89.32(2.05)	
Marital status, n (%)				0.01
Solitude	29.05(0.02)	30.37(1.97)	22.80(1.79)	
Cohabitation	70.95(0.04)	69.63(1.97)	77.20(1.79)	
BMI, n (%)				0.01
<25kg/m2	30.31(0.01)	31.92(0.94)	22.75(2.26)	
25~30kg/m2	42.39(0.02)	42.60(1.61)	41.42(3.55)	
≥30kg/m2	27.30(0.02)	25.49(1.39)	35.83(3.45)	
FIR, n (%)				0.23
<1.3	15.76(0.01)	15.27(1.22)	18.09(2.53)	
1.3~3.5	34.26(0.02)	33.65(1.59)	37.11(3.98)	
≥3.5	49.98(0.03)	51.08(2.20)	44.80(3.55)	
Race, n (%)				0.48
Non-Hispanic White	75.60(0.04)	75.19(2.48)	77.52(4.07)	
Non-Hispanic Black	8.76(0.01)	8.94(1.21)	7.89(1.66)	
Mexican American	7.54(0.01)	7.88(1.00)	5.90(1.11)	
Other Race	8.11(0.02)	7.98(2.03)	8.69(4.14)	
Education level, n (%)				0.01
Less than or high school	43.60(0.03)	42.21(1.87)	50.14(3.69)	
Above high school	56.40(0.03)	57.79(1.87)	49.86(3.69)	
Alcohol intaking, n (%)				0.002
No	23.03(0.04)	20.43(3.99)	35.27(3.66)	
Yes	76.97(0.05)	79.57(3.99)	64.73(3.66)	
Smoking status, n (%)				< 0.0001
Never	43.51(0.03)	46.47(2.60)	29.54(2.82)	
Former	29.69(0.02)	26.19(1.50)	46.18(2.31)	
Current	26.80(0.02)	27.34(1.68)	24.28(2.91)	
Vigorous activity				< 0.0001
No	53.52(0.03)	50.88(1.83)	65.93(3.29)	
Yes	44.51(0.02)	48.22(1.76)	27.00(3.25)	
Unable to do activity	1.98(0.00)	0.90(0.29)	7.07(1.90)	
Moderate activity				< 0.0001
No	44.93(0.02)	44.12(2.14)	48.79(3.29)	
Yes	54.11(0.03)	55.64(2.19)	46.89(3.38)	
Unable to do activity	0.96(0.00)	0.24(0.17)	4.33(1.79)	
DM, n (%)				< 0.0001
No	90.90(0.04)	94.79(0.64)	72.57(2.67)	
Yes	9.10(0.01)	5.21(0.64)	27.43(2.67)	
CVD, n (%)				< 0.0001
No	92.20(0.04)	94.97(0.56)	79.13(2.97)	
Yes	7.80(0.01)	5.03(0.56)	20.87(2.97)	
Hypertension, n (%)				< 0.0001
No	68.56(0.04)	73.43(2.09)	45.58(2.58)	
Yes	31.44(0.02)	26.57(2.09)	54.42(2.58)	
CKD, n (%)				< 0.0001
No	89.53(0.04)	93.03(0.53)	73.04(2.11)	
Yes	10.47(0.01)	6.97(0.53)	26.96(2.11)	
Anemia, n (%)				< 0.0001
No	97.36(0.04)	98.27(0.31)	93.06(1.23)	
Yes	2.64(0.00)	1.73(0.31)	6.94(1.23)	
Albumin (g/L)	43.75 ± 0.12	44.01 ± 0.13	42.53 ± 0.13	< 0.0001
CRP(mg.dl)	0.33 ± 0.02	0.31 ± 0.02	0.43 ± 0.04	0.004
Ast(U/L)	26.83 ± 0.58	26.82 ± 0.70	26.89 ± 1.02	0.96
SII	577.18 ± 10.62	568.16 ± 11.43	619.70 ± 17.54	0.02

Values are mean +/- SD (continuous variables) or n% (categorical variables) are weighted.

BMI, Body mass index; FIR, Family income to poverty ratio; DM, Diabetes mellitus; CVD, Cardiovascular disease; CKD, chronic kidney disease; CRP, C-reactive protein; AST, Aspartate aminotransferase.

### The association between LTL and ED

**Figure 2:**
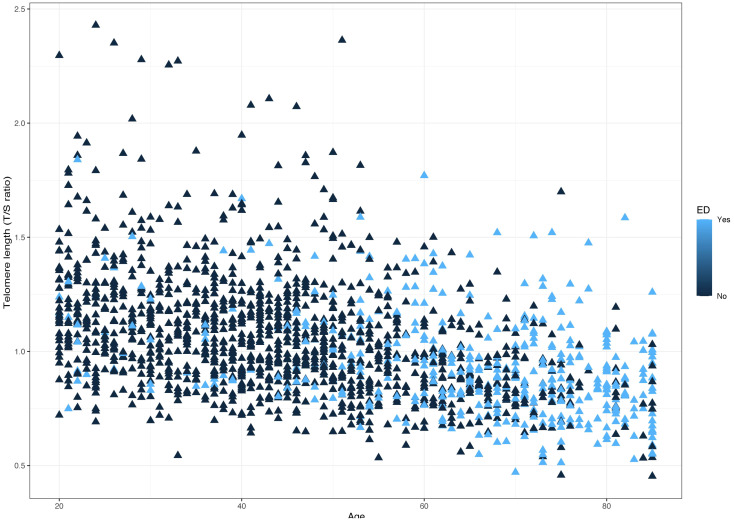
Distribution between age and telomere length (T/S ratio) in ED and non-ED.

**Figure 3:**
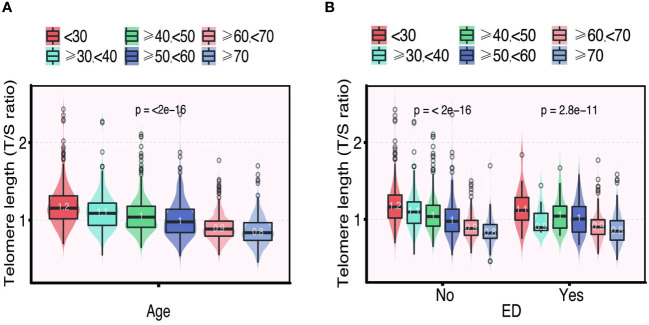
The distribution of telomere length (T/S ratio) grouped by age **(A)**. The distribution of telomere length (T/S ratio) in different age groups categorized by ED **(B)**.

**Figure 4:**
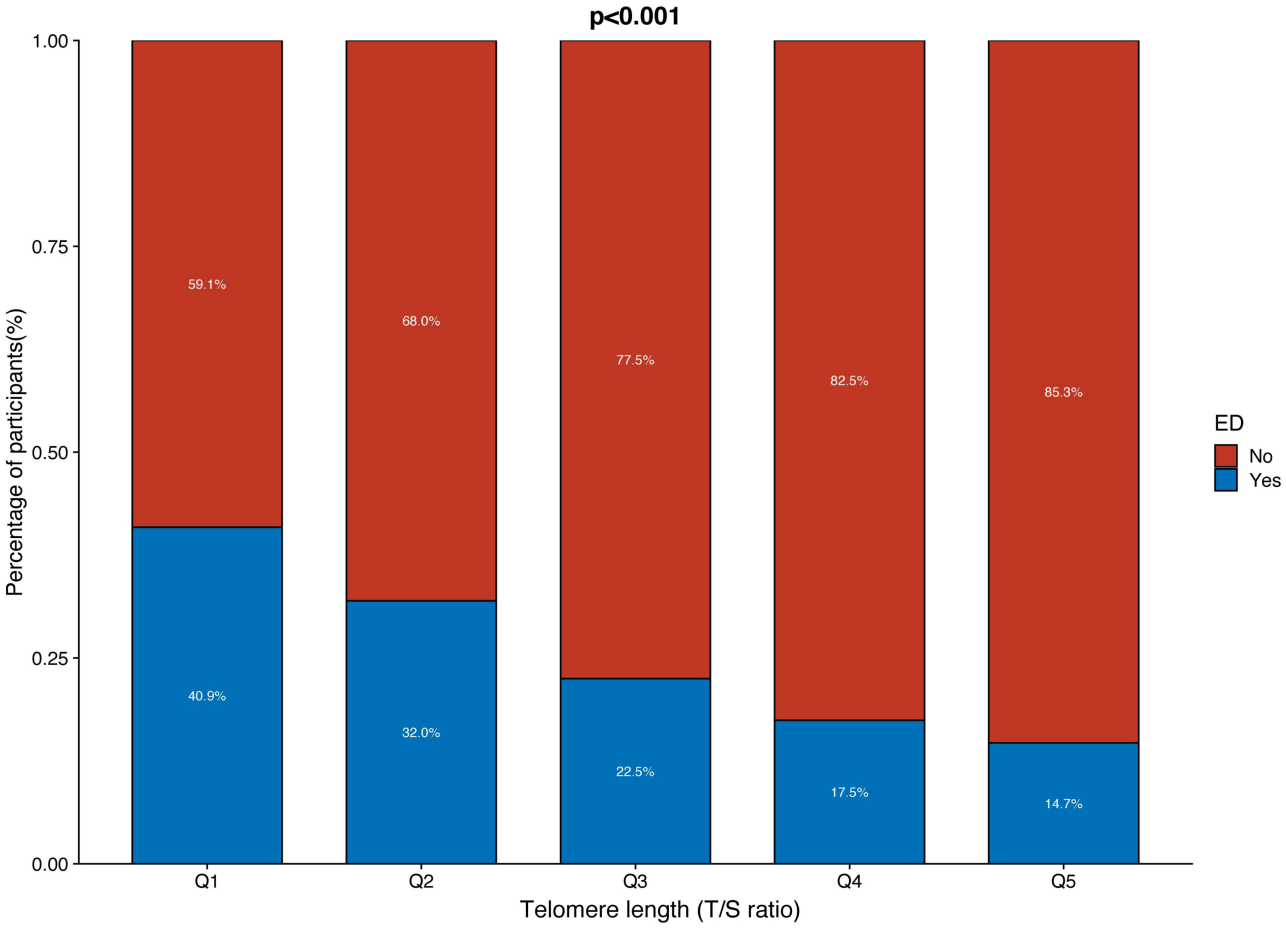
The proportion of ED and non-ED individuals in each quintile of telomere length (T/S ratio).

The distribution of LTL and age in both the ED and non-ED groups is illustrated in **Figure 2**. As age increases, there is a trend towards shorter LTL. Moreover, the distribution of LTL across various age groups is presented. Significant differences in LTL are noted among different age groups (**Figure 3A**, p<0.05), with consistent results observed in both the ED and non-ED groups (**Figure 3B**). Additionally, upon categorizing LTL into quintiles, it is evident that a longer LTL is linked to a reduced incidence of ED (**Figure 4**). The relationship between LTL and ED was assessed in **Table 2** through a weighted multivariable regression analysis. The findings reveal a negative association between LTL and ED. Upon considering LTL as a continuous variable, univariate weighted logistic regression analysis demonstrated that each increase in LTL was linked to an 88% reduction in the risk of ED (OR 0.12, 95% CI 0.06-0.24). This association remained statistically significant even after adjusting for various models (model 1, model 2, model 3). In the fully adjusted model (model 3), each unit increase in LTL corresponded to a 56% decrease in the risk of ED (OR 0.44, 95% CI 0.25-0.79).

**Table 2:** Association between LTL and ED.

Characteristic	Crude model(95%CI)	Model 1(95%CI)	Model 2(95%CI)	Model 3(95%CI)
Continue	0.12(0.06,0.24)	0.31(0.14, 0.67)	0.36(0.20, 0.66)	0.46(0.25, 0.85)
Category				
Q1	ref	ref	ref	ref
Q2	0.70(0.45,1.07)	0.87(0.55, 1.37)	0.93(0.58, 1.47)	1.03(0.69, 1.54)
Q3	0.39(0.28,0.53)*	0.51(0.38, 0.68)*	0.54(0.38, 0.77)*	0.62(0.45, 0.86)*
Q4	0.39(0.24,0.64)*	0.66(0.40, 1.10)	0.77(0.42, 1.39)	0.93(0.49, 1.79)
Q5	0.24(0.16,0.35)*	0.43(0.29, 0.62)*	0.47(0.32, 0.69)*	0.56(0.39, 0.81)*
p for trend)	<0.0001	0.01	0.004	0.03

Unadjusted model: no covariates were adjusted.

Model 1, age, race, marital status, education, FIR and BMI were adjusted.

Model 2, Model 1+alcohol intake, smoking status, vigorous and moderate activity were adjusted.

Model 3, Model 2+,DM, CVD, Hypertension, CKD, Anemia, albumin, CRP, SII, and AST were adjusted.

*: mean p<0.05.

Furthermore, when classifying LTL into five equal categories, the negative relationship between LTL and ED remained consistent. In comparison to the reference group (Q1), the initial analysis showed a 30% decrease in the risk of ED (OR 0.70, 95% CI 0.45-1.07) in the second group (Q2), a 61% decrease (OR 0.39, 95% CI 0.28-0.53) in the third group (Q3), and another 61% decrease (OR 0.39, 95% CI 0.24-0.64) in the fourth group (Q4). The risk of ED was further reduced by 76% (OR 0.24, 95% CI 0.16-0.35) in the fifth group (Q5). After accounting for all variables in model 3, the risk of ED decreased by 38% in the third group (OR 0.62, 95% CI 0.45-0.86), 7% in the fourth group (OR 0.93, 95% CI 0.49-1.79), and 44% in the fifth group (OR 0.56, 95% CI 0.39-0.81).

**Figure 5:**
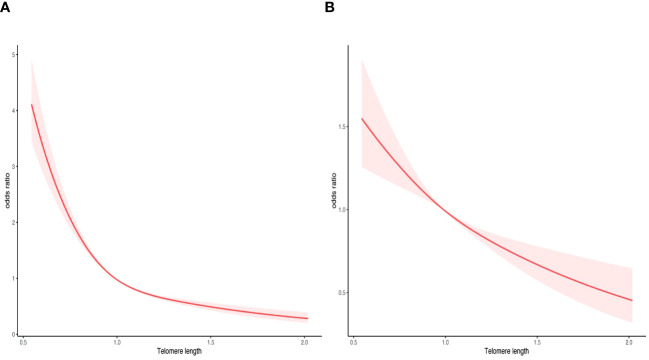
Smooth curve fitting for telomere length and ED. **(A)** crude model. **(B)** adjusted for age, race, marital status, education, FIR, BMI, DM, CVD, hypertension, CKD, anemia, albumin, CRP, SII, and AST. The area between the upper and lower light pink is on behalf of 95% CI. The red solid line indicates that the negative linear association between telomere length and ED is proven by generalized additive model.

Moreover, a RCS analysis was conducted to investigate the potential nonlinear correlation between LTL and ED (**Figure 5**). By employing logistic regression modeling and fitting smooth curves, we observed a nonlinear relationship between LTL and ED in the crude model (**Figure 5A**). However, after controlling for other variables, this nonlinear relationship became insignificant (**Figure 5B**). The data were fitted to a piecewise multivariate logistic regression model, which allowed for two distinct slopes. In our study, the p-value for the log-likelihood ratio test was 0.002 (**Table 3**), therefore we employed a two-piecewise model to establish the association between LTL and ED. We identified an inflection point at approximately 1.14. On the left side of the inflection point, the effect size was 0.05 (0.017, 0.14) in the crude model, 0.17 (0.037, 0.78) in model 1, 0.21 (0.055, 0.79) in model 2, and 0.32 (0.079, 1.33) in model 3. On the right side of the inflection point, the effect size was 0.26 (0.046, 1.44) in the crude model, 0.32 (0.023, 4.50) in model 1, 0.23 (0.038, 1.40) in model 2, and 0.25 (0.038, 1.67) in model 3.

**Table 3:** The nonlinear relationship between LTL and ED.

Telomean length	Crude model (95%CI)	Model 1 (95%CI)	Model 2 (95%CI)	Model 3 (95%CI)	P-value
<1.14	0.050(0.017,0.14)*	0.170(0.037, 0.78)*	0.209(0.055, 0.79)*	0.32(0.079, 1.33)	0.11
>=1.14	0.256(0.046,1.44)	0.322(0.023, 4.50)	0.230(0.038, 1.40)	0.25(0.038, 1.67)	0.14
Likelihood Ratio test					0.002

The bold values and * are combined together, indicating mean p value <0.05.

### Subgroup analysis

To validate the association between LTL and ED, we conducted subgroup analyses to ensure robustness and validity. These analyses were performed within three models (Model 1, Model 2, Model 3), and interaction tests were carried out in Model 3. The comprehensive findings from these subgroup analyses can be found in **Table 4**. Across various subgroups, including age, race, marital status, BMI, education level, alcohol intake, smoking status, CVD, DM, CKD, and hypertension, we consistently observed a significant and consistent relationship between LTL and ED. Importantly, no interactions were detected.

**Table 4:** Subgroup analysis of the association between LTL and ED among U.S. men in the NHANES 2001–2002.

Characteristic	Crude model (95%CI)	Model 1 (95%CI)	Model 2 (95%CI)	Model 3 (95%CI)	p for interaction*
Age					0.26
<40 years	0.16(0.02,1.04)	0.16(0.02,1.46)	0.15(0.01,2.27)	0.15(0.01, 1.54)	
>=40 years	0.30(0.16,0.57)	0.33(0.17,0.62)	0.37(0.16,0.86)	0.50(0.25,1.02)	
BMI					0.31
<25kg/m2	0.05(0.01,0.21)	0.25(0.06, 1.03)	0.28(0.05, 1.47)	0.40(0.10, 1.61)	
25~30kg/m2	0.10(0.04,0.23)	0.20(0.09,0.46)	0.22(0.08,0.63)	0.27(0.11, 0.68)	
≥30kg/m2	0.35(0.08,1.50)	0.56(0.12, 2.55)	0.62(0.11, 3.66)	0.78(0.19, 3.25)	
Race					0.13
Non-Hispanic White	0.10(0.04,0.24)	0.26(0.12, 0.55)	0.31(0.12, 0.80)	0.44(0.20, 0.95)	
Non-Hispanic Black	0.18(0.03,1.17)	0.33(0.04, 2.64)	0.33(0.02, 6.07)	0.38(0.10, 1.50)	
Mexican American	0.32(0.08,1.28)	0.46(0.03,6.51)	0.48(0.11,2.12)	0.65(0.12, 3.45)	
Other Race	0.22(0.03,1.44)	1.02(0.07,14.95)	1.05(0.03, 42.31)	1.85(0.05, 67.77)	
Education level					0.08
Less than or high school	0.22(0.11,0.44)	0.50(0.26, 0.98)	0.55(0.25, 1.23)	0.70(0.34,1.43)	
Above high school	0.07(0.02,0.21)	0.16(0.06, 0.46)	0.18(0.05, 0.61)	0.24(0.08,0.76)	
FIR					0.59
<1.3	0.18(0.05,0.62)	0.37(0.08, 1.72)	0.27(0.06, 1.18)	0.23(0.07,0.70)	
1.3~3.5	0.12(0.04,0.32)	0.32(0.13, 0.76)	0.38(0.14, 1.05)	0.50(0.19, 1.27)	
≥3.5	0.10(0.03,0.41)	0.21(0.06, 0.81)	0.23(0.04, 1.25)	0.38(0.10, 1.45)	
Marital status					0.13
Solitude	0.13(0.04,0.46)	0.59(0.16, 2.11)	0.59(0.14, 2.44)	0.99(0.25, 3.95)	
Cohabitation	0.13(0.06,0.28)	0.25(0.12, 0.50)	0.29(0.11, 0.71)	0.37(0.17,0.83)	
Smoking status					0.79
Never	0.11(0.02,0.47)	0.25(0.07, 0.92)	0.27(0.07, 1.03)	0.30(0.10, 0.97)	
Former	0.24(0.11,0.54)	0.41(0.19, 0.86)	0.43(0.17, 1.09)	0.54(0.20,1.45)	
Current	0.08(0.04,0.17)	0.17(0.05, 0.53)	0.18(0.05, 0.70)	0.22(0.06, 0.87)	
Alcohol intaking					0.15
No	0.28(0.08,1.03)	0.72(0.16, 3.19)	0.71(0.13, 3.76)	0.98(0.15, 6.49)	
Yes	0.10(0.04,0.22)	0.21(0.09,0.49)	0.22(0.09,0.56)	0.30(0.16,0.58)	
CVD					0.49
No	0.13(0.06,0.25)	0.30(0.15, 0.59)	0.32(0.13, 0.78)	0.41(0.20,0.82)	
Yes	0.56(0.12,2.60)	0.66(0.09, 4.64)	0.82(0.07, 8.97)	0.62(0.08, 4.60)	
DM					0.78
No	0.14(0.07,0.27)	0.33(0.18,0.59)	0.36(0.17, 0.75)	0.49(0.25,0.95)	
Yes	0.26(0.05,1.45)	0.28(0.03, 2.58)	0.26(0.01, 5.74)	0.33(0.05, 2.28)	
Hypertension					0.07
No	0.08(0.04,0.17)	0.20(0.08, 0.48)	0.20(0.06, 0.63)	0.26(0.11, 0.58)	
Yes	0.34(0.12,0.96)	0.56(0.20, 1.56)	0.59(0.17, 2.03)	0.70(0.23,2.12)	
CKD					0.73
No	0.14(0.07,0.29)	0.32(0.17, 0.63)	0.36(0.15, 0.86)	0.43(0.20,0.93)	
Yes	0.67(0.11,4.21)	1.00(0.11, 8.63)	1.10(0.08,16.06)	0.50(0.08,3.12)	

Unadjusted model: no covariates were adjusted.

Model 1, age, race, marital status, education, FIR and BMI were adjusted.

Model 2, Model 1+alcohol intake, smoking status, vigorous and moderate activity were adjusted.

Model 3, Model 2+,DM, CVD, Hypertension, CKD, Anemia, albumin, CRP, SII, and AST were adjusted.

Analysis was stratified by age, race, BMI, FIR, education level, alcohol intake, smoking status, vigorous activity, moderate activity, diabetes, CVD, CKD and hypertension, not adjusted for the stratification variable itself.

*means only in model 3.

### Sensitivity analysis

To establish a more rigorous association between leukocyte LTL and ED, we employed a more stringent criterion. Specifically, we only considered individuals who reported never achieving a satisfactory erection. Remarkably, the ORs obtained from Models 1, 2, and 3 exhibited consistent patterns (**Table 5**). Notably, in Model 3, each increment in LTL was associated with a 71% lower risk of ED (OR = 0.29, 95% CI: 0.13-0.65).

**Table 5:** sensitive analysis of association between LTL and ED#.

Characteristic	crude model(95%CI)	Model 1(95%CI)	Model 2(95%CI)	Model 3(95%CI)
Continue	0.05(0.02,0.10)	0.19(0.08, 0.44)	0.20(0.10, 0.40)	0.29(0.13, 0.65)
Category				
Q1	ref	ref	ref	ref
Q2	0.61(0.33,1.11)	0.78(0.45, 1.35)	0.79(0.45, 1.40)	1.08(0.62, 1.90)
Q3	0.45(0.24,0.85)*	0.64(0.38, 1.07)	0.61(0.37, 1.03)	0.85(0.46, 1.60)
Q4	0.18(0.11,0.28)*	0.35(0.20, 0.62)*	0.39(0.22, 0.67)*	0.51(0.26, 0.98)*
Q5	0.23(0.14,0.37)*	0.49(0.28, 0.85)*	0.51(0.28, 0.91)*	0.68(0.38, 1.22)
p for trend	<0.0001	0.01	<0.001	0.04

Unadjusted model: no covariates were adjusted.

Model 1, age, race, marital status, education, FIR and BMI were adjusted.

Model 2, Model 1+alcohol intake, smoking status, vigorous and moderate activity were adjusted.

Model 3, Model 2+,DM, CVD, hypertension, CKD, anemia, albumin, CRP, SII, and AST were adjusted.

# mean ED is defined as never able to keep an erection.

The bold values and * are combined together, indicating mean p value <0.05.

## Discussion

Our study aimed to investigate the potential correlation between the LTL and ED using a nationally representative cross-sectional design. This topic has received limited research attention, highlighting the gap in knowledge that our study addresses. We found a negative correlation between LTL and ED, suggesting that longer LTL are associated with an decreased risk of ED. To account for confounding factors, we conducted multiple logistic regression analysis and various sensitivity analyses, ensuring the stability and robustness of our results. Additionally, this is the first population-based cross-sectional analysis to examine the influence of LTL on ED, adding valuable insights to the existing literature in this field.

Extensive research (21–25) has demonstrated a significant correlation between telomere shortening and the development and progression of aging, cancer, and various diseases. Thorough exploration into the functionality and regulation of telomere holds great promise in enhancing our understanding of its impact on human health and disease. This knowledge creates opportunities for potential future therapeutic interventions.

Previous studies (25–29) have linked shorter LTL to traditional risk factors for ED, such as smoking, diabetes mellitus, cardiovascular disease, hypertension, and obesity. In our study, we collected an extensive range of indicators, including demographic data, lifestyle habits, and medical history, while also considering potential confounding factors related to ED. Consistent results were obtained through the use of diverse adjustment models. Initially, we observed a non-linear relationship between LTL and ED in the unadjusted model. However, this relationship disappeared after accounting for confounding factors. Further investigation is necessary to clarify the nature of this non-linear relationship.

Patients with ED often have multiple chronic conditions (30–32). The impact of LTL on ED may be influenced by different disease states. Subgroup analyses indicated that longer LTL had a more pronounced protective effect against ED in control groups compared to disease groups among individuals with cardiovascular disease, hypertension, and chronic kidney disease. Conversely, individuals with diabetes mellitus showed an opposite trend. No interactions were observed between the subgroups, thus reinforcing the stability of the findings.

To validate the reliability of our findings concerning LTL and ED, we additionally narrowed down the definition of ED as individuals who reported being “never able” to maintain an erection. Remarkably, even after adjusting for different models, the results remained robust and consistent.

Recently, Dilixiati et al. (33) presented a study that closely aligns with our investigation of the relationship between LTL and ED. Their research suggests an inverted J-curve relationship between LTL and ED, which is an intriguing concept. Although our study populations are similar, there are minor differences in the participant selection criteria that may have contributed to the observed variations in outcomes. Our research, in comparison to that of Dilixiati et al., incorporates a broader perspective on the relationship between LTL and ED, including BMI, anemia, albumin, CRP, SII, and AST, which are also known risk factors for ED and thus essential to consider. In addition to the variables included in their study, such as hyperlipidemia and ED medications, our study provides a broader perspective on potential confounding factors. While Dilixiati et al.’s logistic regression analysis initially revealed a negative correlation between LTL and ED in the unadjusted model, this relationship became positive after model adjustment, leading them to propose an inverted J-curve relationship. However, they noted that this curve did not fully explain the results of their multivariate logistic regression analysis. Our findings, on the other hand, consistently demonstrate a negative correlation across all analytical approaches, suggesting a potential L-shaped relationship between LTL and ED, which is also consistent with the results of our logistic regression analysis. This underscores the robustness of our observed association. The methodological differences in covariate adjustment, particularly regarding the treatment of age, may account for the divergent findings between the two studies. We selected age as a categorical variable based on preliminary evidence and clinical observations indicating a nonlinear association with ED. In contrast, Dilixiati et al. treated age as a continuous variable, offering an alternative perspective on the data. The nuanced differences between our results and those of Dilixiati et al. highlight the complexity of the LTL-ED relationship. Our study contributes an additional layer of understanding by suggesting that the method of covariate adjustment can significantly influence the interpretation of outcomes. We appreciate the valuable insights provided by Dilixiati et al. and believe that our findings, while distinct, complement the existing body of knowledge and contribute to a more comprehensive appreciation of the LTL-ED dynamic.

The potential mechanisms underlying the association between LTL and ED may be elucidated from the following perspectives: Firstly, studies (34, 35) indicate that telomerase is closely linked to endothelial cell aging. DNA damage and subsequent telomere attrition are implicated as key factors driving the development of endothelial cell aging in vascular diseases. Consequently, endothelial cell dysfunction resulting from cellular senescence can give rise to various vascular-related disorders, with ED emerging as an early clinical manifestation of subclinical endothelial dysfunction. Secondly, telomere shortening is widely recognized as a hallmark of aging and is intricately intertwined with other aging-related processes (21). The malfunctioning of telomeres can exacerbate these mechanisms and contribute to the occurrence and progression of aging-related diseases, including ED. Thirdly, telomere shortening can potentially increase the risk of oxidative stress and inflammatory responses (36–40), which in turn could have adverse effects on erectile function. This is due to the impact on endothelial cell function, vasodilation, and blood flow regulation, ultimately leading to difficulties in achieving and sustaining an erection. Lastly, telomere shortening may be linked to neuronal activity and signal transmission (41–43), which are essential components in the nervous system’s role in erectile function. Disruption of these neural processes by telomere shortening could further impact erectile function. Considering that LTL is associated with numerous diseases that can impact ED, in clinical settings, strategies aimed at slowing down the rate of telomere shortening hold promise for potentially mitigating the occurrence of ED.

The main strength of this study lies in the reliable data obtained from the NHANES database. The survey employed a rigorous design, reasonable sampling, and a large sample size, enabling the inclusion of abundant variables. Additionally, multiple regression analysis models were constructed to account for various confounding factors during data analysis. Furthermore, different forms of independent variables, including both continuous and categorical variables, were included in the models.

We would like to acknowledge a few limitations in our study that need to be considered. Firstly, the diagnosis of ED relied on self-reported information from the participants, without any clinical confirmation or assessment of the severity of ED. This could potentially introduce misclassification or recall bias. Secondly, due to the cross-sectional design of our study, we are unable to establish causal relationships between LTL and ED. To further investigate the role of LTL in ED and determine the persistence of the associations observed in our study, it is essential to conduct longitudinal studies with larger sample sizes and extended follow-up periods. Thirdly, it is important to highlight that the findings of our study may not directly apply to other racial/ethnic groups or geographical regions, as our study primarily focused on the population of the United States. Despite these limitations, our population-based study significantly adds to our understanding of the relationship between LTL and ED in adults.

## Conclusions

Our study revealed a notable inverse relationship between LTL and the likelihood of ED. Individuals with longer LTL demonstrated a lower risk of developing ED. Therefore, interventions aimed at delaying or counteracting LTL shortening could potentially reduce the risk of ED.

## Data availability statement

The original contributions presented in the study are included in the article/supplementary material. Further inquiries can be directed to the corresponding authors.

## Ethics statement

The studies involving humans were approved by the National Health Statistics Center. The studies were conducted in accordance with the local legislation and institutional requirements. The participants provided their written informed consent to participate in this study. Written informed consent was obtained from the individual(s) for the publication of any potentially identifiable images or data included in this article.

## Author contributions

XC: Writing – original draft. BL: Formal Analysis, Writing – original draft. JZ: Methodology, Writing – original draft. JL: Methodology, Software, Writing – original draft. WJ: Writing – review & editing. RX: Conceptualization, Validation, Writing – review & editing.
